# Immunolocalization of NLRP3 Inflammasome in Normal Murine Airway Epithelium and Changes following Induction of Ovalbumin-Induced Airway Inflammation

**DOI:** 10.1155/2012/819176

**Published:** 2012-03-18

**Authors:** Hai B. Tran, Martin D. Lewis, Lor Wai Tan, Susan E. Lester, Leonie M. Baker, Jia Ng, Monica A. Hamilton-Bruce, Catherine L. Hill, Simon A. Koblar, Maureen Rischmueller, Richard E. Ruffin, Peter J. Wormald, Peter D. Zalewski, Carol J. Lang

**Affiliations:** ^1^Centre for Inflammatory Disease Research (CIDR), The Basil Hetzel Institute for Translational Health Research, Woodville South, SA 5011, Australia; ^2^Stroke Research Programme and Neurology Department, University of Adelaide School of Medicine, The Queen Elizabeth Hospital, Woodville South, SA 5011, Australia; ^3^School of Molecular and Biomedical Science, The University of Adelaide, Adelaide, SA 5005, Australia; ^4^Department of Surgery–Otorhinolaryngology Head and Neck Surgery, University of Adelaide, The Queen Elizabeth Hospital, Woodville South, SA 5011, Australia; ^5^Rheumatology Department, The Queen Elizabeth Hospital, Woodville Road, Woodville South, SA 5011, Australia; ^6^Discipline of Medicine 5B, University of Adelaide, The Queen Elizabeth Hospital, Woodville South, SA 5011, Australia

## Abstract

Little is known about innate immunity and components of inflammasomes in airway epithelium. This study evaluated immunohistological evidence for NLRP3 inflammasomes in normal and inflamed murine (Balb/c) airway epithelium in a model of ovalbumin (OVA) induced allergic airway inflammation. The airway epithelium of control mice exhibited strong cytoplasmic staining for total caspase-1, ASC, and NLRP3, whereas the OVA mice exhibited strong staining for active caspase-1, with redistribution of caspase-1, IL-1**β** and IL-18, indicating possible activation of the NLRP3 inflammasome. Active caspase-1, NLRP3, and other inflammasome components were also detected in tissue eosinophils from OVA mice, and may potentially contribute to IL-1**β** and IL-18 production. In whole lung, inRNA expression of NAIP and procaspase-1 was increased in OVA mice, whereas NLRP3, IL-1**β** and IL-18 decreased. Some OVA-treated mice also had significantly elevated and tightly correlated serum levels of IL-1**β** and TNF**α**. In cultured normal human bronchial epithelial cells, LPS priming resulted in a significant increase in NLRP3 and II-lp protein expression. This study is the first to demonstrate NLRP3 inflammasome components in normal airway epithelium and changes with inflammation. We propose activation and/or luminal release of the inflammasome is a feature of allergic airway inflammation which may contribute to disease pathogenesis.

## 1. Introduction

Asthma affects up to 12% of adults and 25% of children in Australia and there is a significant undiagnosed cohort [1]. Although we have a range of medications that are effective in their own right, there is a need to further improve the management of this disease. This involves better clinical programs and an improved understanding of the basic mechanisms of asthma so that new ways can be introduced which work synergistically with conventional asthma medications to modify airway inflammation.

Asthma is a disease characterized by both bronchiolar smooth muscle constriction and a chronic airway inflammation. Some of the features of the disease are modelled in the well-characterized acute and chronic models of OVA-induced allergic airway inflammation in mice [2, 3]. The inflammatory component of asthma is generally thought of as a Th2-driven process, involving eosinophil recruitment to the airways and consequent damage, including airway epithelial cell death [4]. Damage and repair eventually lead to a remodelling of the airways which increases the sensitivity of the airway muscle to cholinergic agonists and further restricts airflow.

 However, other inflammatory signalling pathways may also play a significant role in the pathogenesis and progression of the disease. Of particular interest is the role of the IL-1 family of cytokines that are key components of the innate immune response [5]. IL-1*β* and IL-18 are activated by cytosolic multiprotein complexes called inflammasomes [6, 7]. Inflammasomes have been best characterized in the monocyte-macrophage cell lineage, but recent evidence indicates that gingival (and perhaps other) types of epithelial cells may also contain these structures [8]. Their generic structure includes (i) a member of the nucleotide-binding oligomerization domain- (NOD-) like receptor (NLR) family of pattern recognition molecules specific for each type of inflammasome, (ii) apoptosis-associated speck-like protein containing a caspase-recruitment domain (ASC), and (iii) caspase-1. Other inflammatory caspases or caspase-regulatory molecules such as X-linked or neuronal inhibitor of apoptosis proteins, XIAP and NIAP, respectively, may also be recruited. Different types of inflammasome (e.g., Nlrp1, Nlrp3, IPAF, and AIM2) have been identified based on the NLR component (or the non-NLR equivalent) which forms the complex. Of these the best characterized is the Nlrp3 inflammasome which plays a predominant role in IL-1*β* and IL-18 production [7]. In macrophages, IL-1*β* production, processing, and release requires the interaction of two signalling pathways. Binding of ligands such as lipopolysaccharide (LPS) to membrane toll-like receptor 4 (TLR-4) triggers the synthesis of pro-IL-1*β* while a number of danger signals including molecules released from necrotic cells (e.g., ATP and uric acid) promote assembly of the Nlrp3 inflammasome complex, activation of caspase-1 from its precursor, processing of IL-1*β* to its active form, and release of IL-1*β* from the cells [8].

Inflammasome involvement in asthma inflammation is a relatively new concept. An early protective role for inflammasomes might be predicted by the “hygiene hypothesis”, whereby exposure to microbes and their products (such as LPS) early in life is thought to protect against development of asthma, perhaps by a skewing of the immune response away from one dominated by Th2 cytokines [9]. However, current evidence would favour a proinflammatory role for IL-1*β* since (i) there are increased levels of serum, BALF, and bronchial epithelial IL-1*β* in human asthmatics, compared to healthy subjects [10–12], (ii) increase in serum IL-1*β* has also been reported in primates [13], (iii) IL-1*β* levels were decreased 2-fold in the bronchial epithelium following inhalation of beclomethasone dipropionate (as measured by an immunohistological technique) [14], and (iv) administration of TNF-*α* and IL-1*β* induces airway hyper-reactivity, a feature of asthma [15, 16]. IL-18, another potent pro-inflammatory cytokine whose maturation requires activation of caspase-1 on the inflammasome, is typically considered as a Th1 cytokine due to its effects associated with IFN-*γ*. Increased serum IL-18 has also been described in asthmatics [17–19]. Finally, danger signal molecules such as uric acid and extracellular ATP have been shown to mediate inflammasome-dependent inflammation in experimental rodent models of lung injury [20] and asthma [21], respectively.

In addition to providing a physical barrier to inhaled pathogens, allergens, and other foreign agents, the airway mucosa (epithelium and secretions) has a number of potential mechanisms by which it can contribute to innate immune defences in the lung. Loss of the integrity of the airway epithelium is widely thought to be a critical component in the pathogenesis of asthma [22]. To our knowledge, there has been no systematic study of the inflammasome in airway epithelium. In this study, we have looked for evidence of Nlrp3 inflammasome involvement in OVA-induced airway inflammation in mice, as a model of human asthma, as well as in primary bronchial epithelial cultures.

## 2. Materials and Methods

### 2.1. Antibodies

Rabbit polyclonal antibodies (Abs) to IL-1*β*, Nlrp3, total caspase-1; goat Abs to caspase-1 P10 and P20 active subunits; and mouse monoclonal Ab (mAb) to SP-D were from Santa Cruz Biotechnology (Santa Cruz, CA, USA). Other rabbit Abs were ASC from Abcam (Cambridge, UK), CCR3 from Epitomics (Burlingame, CL, USA), and IL-18 from Rockland (Gilbertsville, Pennsylvania, USA). Mouse mAb to human NLRP3 was from Enzo (Enzo Life Sciences Inc., Farmingdale, NY, USA). Secondary Abs were sheep F(ab′)2 anti-rabbit IgG (Cy3), goat F(ab′)2 anti-rabbit IgG (FITC), rabbit anti-goat IgG (Cy3), sheep F(ab′)2 anti-mouse IgG (Cy3), (Sigma-Aldrich Chemicals, St Louis, MO, USA), and a donkey F(ab′)2 anti-mouse IgG DyLight594 (Jackson ImmunoResearch, West Grove, PA, USA).

### 2.2. Mouse Model of Allergic Airway Inflammation

A classical mouse model [23] that induces strong eosinophilic inflammation, airway hyper-responsiveness, and vascular and parenchymal changes after sensitisation and challenge with OVA was used to induce inflammation. All experiments were performed under the University of Adelaide Animal Ethics Committee approval number M-57-2007, and in compliance with “Principles of Animal Care” publication number 86-23 of the National Institute of Health and the “Australian Code of Practice for the Care and Use of Animals for Scientific Purposes”, 6th Edition.

Female Balb/c mice (age 4–6 weeks; specific pathogen free) were purchased from the University of Adelaide, Adelaide, Australia. Three mice were set aside for analysis of inflammasome components in the absence of any stimulus. The remaining mice were divided into two experimental groups (*n* = 8 in each group) and housed in plastic cages (38 × 25 cm) at 21°C with a 14 h light/10-h dark cycle. OVA-treated mice received 50 *μ*g of chicken OVA in 1 mL of alhydrogel (CSL, Parkville, Australia) in 0.9% sterile saline, i.p. on days 0 and 14. Control (SAL) mice received alhydrogel in 0.9% saline alone. Mice sensitized to OVA were then aerochallenged with 10 mg/mL OVA in 0.9% saline from day 22 to day 32, for 30 min, three times a day, every 2nd day, using a side-stream nebulizer, which produced particles of 1–3 *μ*m (Fisher and Paykel, Sydney, Australia). SAL-treated groups were nebulised with 0.9% saline alone.

### 2.3. Collection of Tissues

Mice were bled from the tail for preparation of sera. For tissue collection, mice were anaesthetized by an i.p. dose of pentobarbitone sodium (50 mg kg^−1^), 22 h after the last nebulization. The trachea was cannulated with a blunted 19-gauge needle and bronchoalveolar lavage fluid (BALF) was collected by lavaging the lungs three times by instilling and withdrawing the same volume (1 mL) of ice-cold Hanks buffered salt solution (HBSS, pH 7.4). One part of the BALF sample was immediately cytospun onto slides and the other part was centrifuged to obtain supernatant for cytokine and uric acid assay. Mice were then sacrificed and other tissue samples collected. Tissue samples were archived as appropriate for various downstream measurements, including standard assessments of airway and tissue eosinophilia and lung histopathology.

### 2.4. Isolation of Lung mRNA and Reverse Transcription

Samples of mouse lung tissue were immediately placed into RNA later (Applied Biosystems/Ambion, USA). Tissue was homogenized using a TissueRuptor (Qiagen Pty Ltd, Australia) and RNA extracted using an RNeasy Mini Kit (Qiagen Pty Ltd, Australia). An on-column DNAse treatment was performed using an RNase-Free DNase Set (Qiagen Pty Ltd, Australia). RNA quality was checked using an Experion RNA StdSens Kit on the Experion Electrophoresis station (BioRad Laboratories Inc, Australia). Mouse RNA samples obtaining RQI of 7–10 (green) were used in the subsequent array analysis. RNA content was quantified using a Nano Drop 1000 Spectrophotometer (Thermo Scientific, USA). 1000 ng of RNA was reverse transcribed into cDNA using the RT^2^ First Strand Kit (Qiagen Pty Ltd, Australia). The incubation steps for the reaction were performed on a MyCycler Thermal Cycler (BioRad Laboratories Inc, Australia).

### 2.5. Real-Time Quantitative PCR Array

Mouse cDNA samples were combined with RT^2^ SYBR Green/Fluorescein qPCR Master Mix with Nuclease-free Sterile H_2_O (Qiagen Pty Ltd, Australia) and then added across an entire RT2 Profiler PCR Mouse Inflammasome Array (PAMM-097A) 96-well plate. Each mouse sample (*n* = 8 from each group) was tested on a separate 96-well array plate containing a panel of 84 wells pertaining to different genes of the inflammasome pathway, 5 house keeping control wells, 1 genomic DNA control well, 3 reverse transcription control wells, and three positive PCR control wells. PCR was performed using an iCycler with iQ5 Software (BioRad Laboratories Inc, Australia) and Cq data was then analysed by the ΔΔCt method using the RT^2^ Profilier Array data spreadsheet (Qiagen Pty Ltd, Australia).

### 2.6. Measurement of Cytokines

Serum cytokines were measured by Bioplex as per the manufacturer's instructions (BioRad Laboratories Inc, Australia). In brief, the concentration of total protein per sample was determined by Mini Bradford assay (BioRad Laboratories Inc, Australia) using standards of bovine serum albumin (Sigma-Aldrich Chemicals, St Louis, MO, USA). Samples were normalised to 100 *μ*g total protein and analysed in duplicate using a Bioplex Promouse Cytokine 8 plex magnetic bead array. All wash steps were performed using the Bioplex II Wash Station. Plate data was collected using a Bioplex 200 suspension array system and analysed using the Bioplex Manager 5 Software (BioRad Laboratories Inc, Australia).

### 2.7. Immunofluorescence Studies

Paraffin tissue sections of 5 *μ*m thickness were treated for antigen retrieval by microwave heating in 10 mM citrate buffer pH 6.0 unless otherwise stated. Tissue sections were also heated in 10 mM Tris EDTA buffer pH 9.0 for costaining of SP-D with caspase-1, and digested with 1 mg/mL Proteinase K (Promega, Fitchburg, Wisconsin, USA) for staining of P10 and P20 caspase-1 subunits. Sections were blocked with a serum-free protein blocking solution (DakoCytomation Inc., Carpinteria, CA, USA), incubated overnight at 4°C with primary Abs, and 1 h at room temperature with secondary Abs. Immunofluorescence was detected and imaged with a Zeiss microscope equipped with HBO 100 illuminating system, AxioCam MRn digital camera and AxioVision 4.8.1 software (Carl Zeiss GmbH, Goettingen, Germany). 

### 2.8. TUNEL Staining of Cell Death

Cell death was detected in situ using a fluorescence TUNEL kit from Promega (Fitchburg, Wisconsin, USA) following the manufacturer's protocol. 

### 2.9. Measurement of Uric Acid in BALF

Uric acid was measured in BALF according to manufacturer's instructions using the Amplex Red Uric Acid/Uricase Assay Kit (A22181, Invitrogen Aust Pty Ltd, Mulgrave, Australia). Fluorescence measurements were made using a fluorescence plate reader (Optima Fluostar, BMG Labtech, Gmbh, Offenburg, Germany) with excitation at 530 nm and emission read at 590 nm.

### 2.10. Normal Human Bronchial Epithelial Cell Culture

Normal human bronchial epithelial (NHBE) cells and Bronchial Epithelial Growth Medium (BEGM) were obtained from Lonza (Lonza Australia Pty Ltd, Mt Waverley, VIC, Australia). Cells were cultured in BEGM until use at passage 4. Cells at 60–70% confluence were stimulated for 18 h with *E. coli* LPS (5 *μ*g/mL,Sigma-Aldrich Chemicals, St Louis, MO, USA), IFN-*γ* (50 ng/mL, Sigma-Aldrich Chemicals, St Louis, MO, USA), or combination of these two, in 8-well chamber slides (BD Biosciences, Franklin Lakes, NJ, USA). Cells were washed twice with phosphate-buffered saline (PBS) and then fixed with 2.5% formalin/PBS for 10 minutes. Cells were washed 4 times with TBST (tris-buffered saline, pH7.5 added with 0.05% Tween-20), air-dried overnight at room temperature, and stored at −20°C until use.

### 2.11. Immunofluorescence of NHBE

Fixed cells were permeabilized 5 minutes with 1% SDS/PBS at room temperature, then washed 5 times with TBST to remove SDS. Following 1 h blocking incubation with a serum-free protein blocker (DakoCytomation Inc., Carpinteria, CA, USA), cells were incubated with a mixture of primary antibodies (see antibody section of methods) including mouse monoclonal antibody to human Nlrp3 and a rabbit polyclonal anti-human IL-1*β*, overnight at 4°C. Next, following 5 washes with TBST, cells were incubated 1 h at room temperature with a mixture of secondary antibodies including a donkey F(ab′)2 anti-mouse IgG DyLight594 and goat F(ab′)2 anti-rabbit IgG-FITC. Slides were washed 5 times with TBST before cover-slipping and imaging.

For quantitation of fluorescence intensity, multiple microphotos were acquired randomly under a 20x objective. Images were then blinded and analysed using a morphometric software package (ImageJ, NIH, Bethesda, MD, USA).

## 3. Results

### 3.1. Parameters of Airway Inflammation

In keeping with previous descriptions of the acute allergic airway inflammation model [2, 23], the airway inflammation in OVA-challenged mice was characterized by dense peribronchial and perivascular infiltrates of leucocytes (mostly eosinophils) and oedema of bronchial epithelium. Epithelial swelling was more prominent in large bronchi that were sometimes occluded by thickened epithelium. There was swelling of endothelial cells in some blood vessels. No increase of mast cells was detected. None of the saline- (SAL-) treated mice showed any notable histological changes. Typically, there was a 7-fold increase in total BALF cell count from 15.6 ± 11.1 × 10^4^ (median ± 2.5% CI) cells/mL in SAL controls to 117.7 ±  82.7 × 10^4^ cells/mL in OVA-treated mice (*P* < 0.0001). This was largely due to eosinophilia (>85%), although numbers of neutrophils, monocytes, and lymphocytes were also significantly increased (*P* < 0.05, data not shown).

### 3.2. Presence of Inflammasome Components in Normal Mouse Airway Epithelium

To test the hypothesis that the normal airway epithelium possesses the ability to assemble inflammasome complexes, we used immunofluorescence to analyse formalin-fixed lung tissue sections obtained from healthy Balb/c mice for expression of inflammasome common components Nlrp3, ASC and caspase-1, and the substrate cytokines IL-1*β* and IL-18. The same results were obtained for completely untreated mice (naive mice, *n* = 3) and SAL mice (*n* = 8) that were sham-treated with saline i.p. injections and saline nebulisation and used as controls in the experimental murine airway inflammation model.

**Figure 1:**
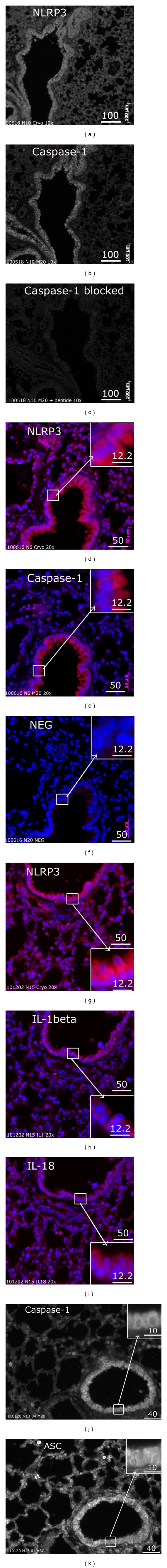
Expression of inflammasome proteins and the IL-1 cytokines in normal mouse lung epithelium. (a–c) Serial sections showing positive labelling for Nlrp3 (a) and total caspase-1 (b) in epithelia of three bronchioles; labelling for caspase-1 was blocked by specific peptide. No blocking peptide for Nlrp3 was available. (d–k) Serial sections showing positive and colocalized labelling of Nlrp3 and caspase-1 (d-e, red), Nlrp3, IL-1*β*, and IL-18 (g–i, red), and caspase-1 and ASC (j-k). (f) Negligible fluorescence was detected using normal rabbit IgG as a negative control for Nlrp3 and all other rabbit antibodies employed in this panel. Blue is DAPI counterstaining of nuclei. Images are representative of similar results from 8 control mice challenged with saline and 3 naive mice. The scale bars are in microns.

**Figure 2:**
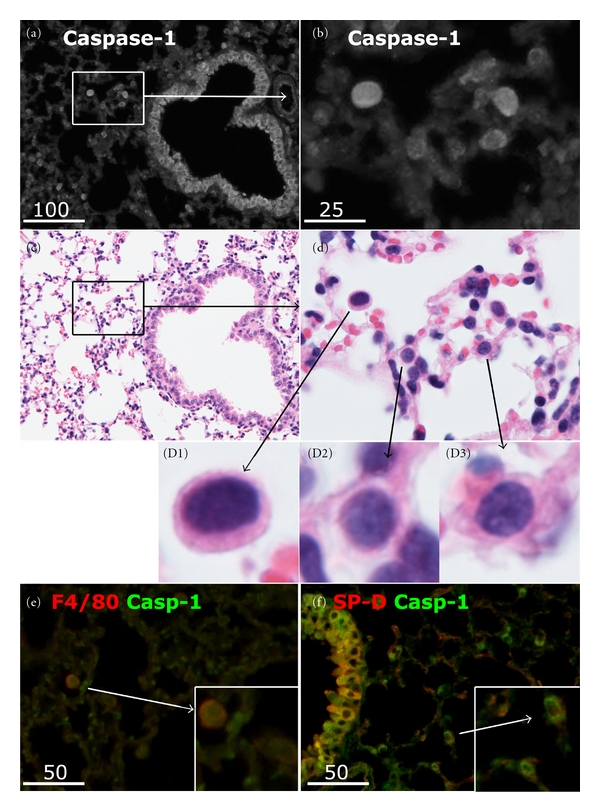
Identification of cell types expressing caspase-1 in normal mouse lung. (a) Representative immunofluorescence image of a normal lung section showing total caspase-1 localized to bronchiolar epithelium as the most distinct site, as well as cells scattered among alveoli (b). (c) and (d) Corresponding images of (a) and (b) following restaining with H&E. Caspase-1 positive cells included alveolar macrophages (D1) or type II alveolar cells, typically found at the alveolar interseptal junctions and containing vacuole-like lamellar bodies in the cytoplasm (D2, D3). (e) Dual labelling of a lung section for the macrophage marker F4/80 (red) and total caspase-1 (green), revealing colocalization (yellow) in macrophages (inset). (f) Dual labelling of a lung section for surfactant protein D (SPD, red) and caspase-1 (green), revealing colocalization in type II alveolar cells (inset). Scale bars are in microns.

Serial sections of control mouse lungs (*n* = 8) were examined by immunohistological labelling for caspase-1 and various other inflammasome-related proteins. Figures 1(a)–1(c) show colocalization of caspase-1 and Nlrp3 in the epithelia of three bronchioles. The fluorescence staining for caspase-1 could be specifically blocked with the relevant immunogen peptide (Figure 1(c)). Blocking peptides were not available for the other antibodies in this panel. However, omission of primary antibody or section incubation with normal rabbit IgG (at matched IgG concentrations) resulted in negligible fluorescence (Figure 1(f)). Colocalization of caspase-1 and Nlrp3 with IL-1*β*, IL-18, and ASC in bronchiolar epithelia was shown (Figures 1(d)–1(k)). There was also colocalization in certain cells scattered among alveoli. It should be noted that the antibodies to caspase-1, IL-1*β*, and IL-18 did not distinguish between their precursor and mature forms. After performing immunofluorescence, some sections were washed and restained with hematoxylin and eosin (H&E) to examine morphology. Figures 2(a) and 2(b) show low and high magnification images of a section of the normal mouse lung immunolabelled for caspase-1, while Figures 2(c) and 2(d) show the corresponding H&E restaining. The caspase-1 positive-staining cells around the alveoli were identified as both alveolar macrophages localised in the alveolar air space (e.g., arrowed cell in Figure 2(D1)) and type 2 alveolar cells (ATII) typically seen at alveolar junctions and showing lamellar bodies in the cytoplasm (Figures 2(D2) and 2(D3)). The identity of these two cell types was confirmed by dual labelling of caspase-1 with either F4/80 (a marker of mouse macrophages, Figure 2(e)) or SP-D (a marker of ATII, Figure 2(f)).

Thus, the normal murine airway epithelium (bronchial epithelium and ATII) expresses, at the protein level, inflammasome components Nlrp3, ASC, and caspase-1, as well as the substrate cytokines IL-1*β* and IL-18.

### 3.3. Presence of Active Caspase-1 in Inflamed, but Not Normal, Mouse Airway Epithelium

**Figure 3:**
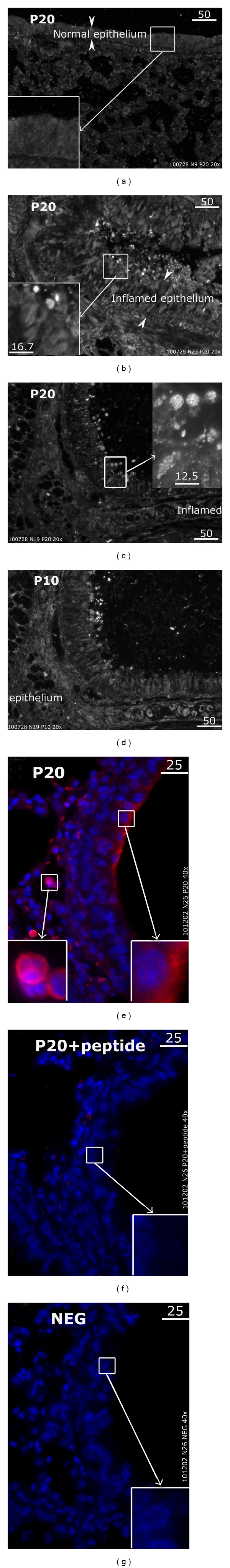
Activation of caspase-1 in inflamed airway epithelium of OVA-treated mice. Antibodies specific to cleaved ends of caspase-1 P20 or P10 subunits were employed to detect activation of caspase-1 in mouse lung tissue. (a) The normal airway epithelium typically expressed little active caspase-1. (b) In the inflamed airway, the swollen epithelium (arrowheads) labelled positively for active caspase-1 as shown by the punctate fluorescence of P20 near the apical surface. (c) and (d) Adjacent serial sections of an inflamed lung stained for antibodies to P20 and P10, respectively, revealed the same punctate patterns of caspase-1 activation near the apical surface. (e–g) In serial sections of an inflamed lung, fluorescence of P20 active caspase-1 antibody (e, red) was inhibited nearly completely by preabsorption of the antibody with the relevant immunogen peptide (f), to a level comparable to that of the conjugate alone control (g). Blue: DAPI counterstaining of nuclei. Images (a-b) are representative of results from P20 staining experiments on multiple sections obtained from 8 control saline-treated mice and 8 OVA-treated mice. Colocalization of P20 and P10 staining (c-d) and P20 peptide blocking experiments (e-f) were carried out on serial sections obtained from 4 inflamed lungs. Scales are in microns.

The antibody for caspase-1 used in the previous series of experiments did not differentiate between the inactive precursor and active form. Using antibodies specific for the cleaved ends of P10 and P20 subunits of caspase-1, we were able to demonstrate very low levels or absence of active caspase-1 in the control mouse lung epithelium (Figure 3(a)). In contrast, distinct patterns of active caspase-1 staining were detected in the epithelium of the inflamed airways of OVA-treated animals (Figures 3(b)–3(d)). In adjacent serial sections of inflamed lung, the two different antibodies P10 and P20 revealed the same punctate patterns of caspase-1 activation near the epithelial apical surface (Figures 3(c) and 3(d)). Preabsorption with the relevant immunogen peptides reduced the labelling of active caspase-1 in the inflamed airway epithelium to a level comparable to that of the conjugate alone (Figures 3(e)–3(g)). Because there are inherent problems in trying to quantify actual changes in fluorescence intensity where there is a redistribution of the label within the treatment accompanying the induction of inflammation, we have not attempted to quantify changes in intensity of immunofluorescence for the various proteins in these sections.

These results suggest that inflammation of the airways in mice is accompanied by conversion of zymogen caspase 1 to its active form and a more apical distribution of inflammasome proteins.

### 3.4. Translocation and Luminal Shedding of the IL-1 Cytokines and Inflammasome Proteins in Allergen-Induced Inflamed Airway Epithelium

**Figure 4:**
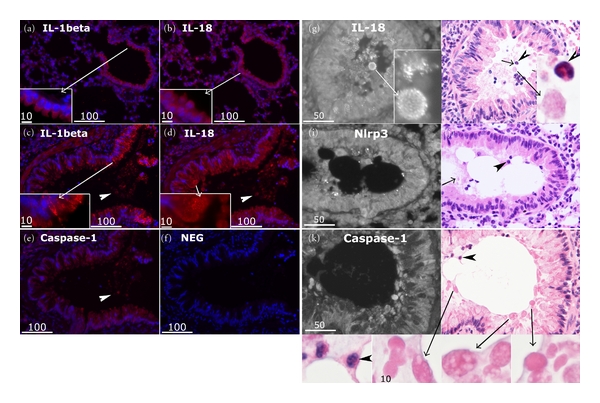
Translocation and luminal shedding of the IL-1 cytokines and inflammasome proteins in inflamed airway epithelium. (a–f) Serial sections of a control mouse lung (a-b) and those of an OVA-treated mouse (c–f) were stained for the cytokines and total caspase-1 in red (Cy3), and counterstained for nuclei with DAPI (blue). In the control mouse lung epithelium, IL-1*β* (a), IL-18 (b), and total caspase-1 (not shown) typically gave an homogenous cytoplasmic fluorescence (*insets*). In staining of multiple sections from the lung of OVA-treated mice, punctate fluorescence of both cytokines was consistently detected near the apical surface (c-d,* insets*). Together with caspase-1, these cytokines were detected in the lumen (c, d, e,* arrowheads*). (f) Control section incubated with the secondary antibody alone. Fluorescence images are representative of similar results from 3 experiments on 8 control and 8 OVA-treated mice. The scale bars are in microns. (g–i) Fluorescently labelled sections of inflamed lungs (g, i, k) were restained with H&E (h, j, l) to identify luminal structures containing inflammasome components and cytokines. Although some fluorescence was localized to infiltrating eosinophils (arrowheads) or cell-free debris (short arrows), most of the luminal shed material appeared to be within well-preserved eosinophilic vesicles (~6–10 microns) near the airway epithelial apex (inset long arrows). H&E images are representative of results from experiments carried out on multiple sections of 4 OVA-treated mouse lung. The scale bars are in microns.

As mentioned earlier, our antibody panel did not differentiate between the inactive precursors and the mature (active) forms of IL-1*β* and IL-18 cytokines. However, there were different patterns of IL-1*β* and IL-18 distribution demonstrated by immunofluorescence between the epithelia of OVA-treated and control mice (Figure 4). In the healthy epithelium, both cytokines distributed more or less homogeneously in the cytoplasm (Figures 4(a) and 4(b)). In contrast, in the inflamed airways, the immunofluorescence of the IL-1 cytokines had a more speckled appearance at the apical surface of the epithelium (Figures 4(c) and 4(d) arrows). Furthermore, immunofluorescence of both IL-1*β* and IL-18 could be detected in the lumen (Figures 4(c) and 4(d), arrowheads). Similarly, luminal staining near the apical surface was also detected for caspase-1 (Figures 4(e) and 4(k)), Nlrp3 (Figure 4(i)), and ASC (not shown). When labelled sections of inflamed mouse lungs were restained with H&E (Figures 4(g)–4(l)), part of the luminal fluorescence of the cytokines and inflammasome proteins was localized to infiltrating leukocytes (mostly eosinophils) or well preserved cell bodies (~6–10 *μ*m in diameter) often seen embedded in the mucus near the epithelial apex (Figure 4(h) and 4(l) insets). It is unlikely that the luminal staining was an artefact caused during tissue processing. Tissues were immediately fixed in formalin for paraffin embedding and the luminal staining was consistently observed in tissue sections, including serial adjacent sections (e.g., Figures 3(c) and 3(d), Figures 4(c)–4(e), with Figure 4(f) as negative control), in all of the mice tissues analysed.

### 3.5. Inflammasome Components in Other Relevant Airway Tissues of Mice with Airway Inflammation

Immunostaining of consecutive adjacent tissue sections with caspase-1 and CCR3, a marker of eosinophils, showed colocalization of CCR3 with caspase-1, detected by antibodies to either the total or the active forms of the caspase (not shown). As CCR3 is not a specific marker of eosinophils but can also be expressed in some other cell types, immunolabelled sections were restained by H&E to identify the type of infiltrating cells positively stained for inflammasome components. In accordance with our previous results >80% of these cells were eosinophils which infiltrated both the submucosal tissue and the airway lumen (Figures 4(h)–4(l)).

Another cell type which might contribute to inflammasome activation in the OVA-induced airway inflammation model is the vascular endothelium. Whereas in saline-treated mice the vascular endothelium stained weakly for total caspase-1, it often stained strongly in the lungs of OVA-treated mice (data not shown). Unfortunately, we could not apply the antibodies to active subunits of caspase-1 to study activation of endothelial caspase-1, due to the nonspecific binding of these antibodies to red blood cells, which was not blocked by the specific peptides (data not shown). The expression of total caspase-1 protein in inflamed but not healthy endothelium was completely removed by blocking with specific peptide (data not shown).

### 3.6. Effects of Mouse Airway Inflammation on Whole Lung Gene Expression

To look at the effect of airway inflammation on expression of gene products with relevance to the inflammasome, we analysed mRNA from lungs of SAL and OVA-treated mice by superarray (Table 1). Typically, there were large increases (8–10 fold) in chemokines (Ccl12, Ccl7, and Cxcl3) known to be important in the mouse airway inflammation model. There were also significant increases with OVA-treatment in expression of a Th2 cytokine IL-6. For the IL-1-like family members, there was a 2.5-fold increase in gene expression for IL-33 and 2- and 11-fold decreases in those for IL-1*β* and IL-18, respectively.

Of the gene products involved in inflammasome regulation, the largest change was an 8-fold increase in the neuronal inhibitor of apoptosis protein NAIP, followed by an almost 4-fold decrease in Nlrp3 expression. Small significant increases were seen for ASC, caspase-1, and AIM2. There were no significant changes in expression of Nlrp1 and Nlrp4 (Table 1).

### 3.7. Cell Death and Uric Acid Levels in Airway Epithelium

**Figure 5:**
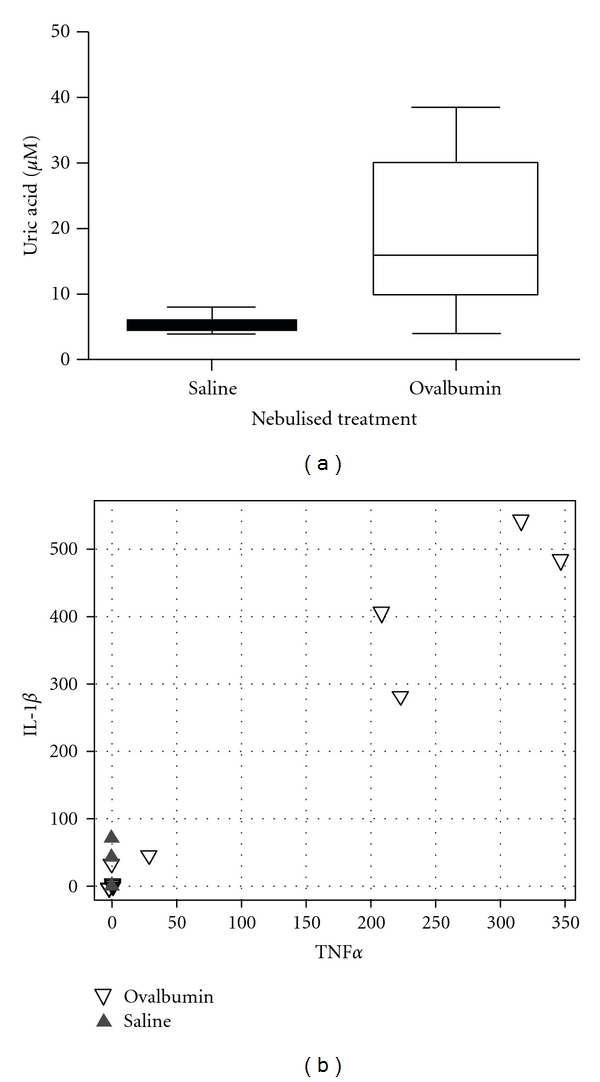
Increase of uric acid in BALFs (a) and cytokines IL-1*β* and TNF-*α* in sera (b) of OVA-treated mice, compared to saline-treated controls. (a) Box plot (showing median, interquartile range, and nonoutlier range) demonstrating that BALF uric acid levels are elevated in OVA-treated mice (*n* = 8) compared to SAL mice (*n* = 8), *P* < 0.001 (Kruskal-Wallis test). (b) Scatter plot of serum IL-1*β* versus TNF-*α* levels (pg/mL) in OVA-treated mice (open triangles, *n* = 8) versus SAL-treated mice (closed triangles, *n* = 8). High cytokine levels were only observed in the OVA-treated mouse group.

In accordance with our previous data [23], using the TUNEL assay we confirmed an increased cell death in the lung sections of OVA- versus SAL-treated animals (data not shown). An analysis of BALF in the asthmatic mice demonstrated a significant increase in uric acid (Figure 5(a)), which is known as both a product of nucleic acid catabolism in dead cells and a stimulus for Nlrp3 inflammasome activation [20]. 

### 3.8. Cytokines in BALF and Serum

We were unable to detect IL-1*β* and IL-18 in the BALF of either saline- or OVA-treated mice (data not shown). This may be due to excessive dilution of the BALF during the lavage or choosing the wrong time-point (22 hr after final challenge) for lavaging.

Analysis of the sera showed high levels (>200 pg/mL) of IL-1*β* in 4 of the 8 OVA-treated mice but in none of the 8 saline-treated control mice. The serum levels of IL-1*β* were tightly correlated with those of TNF-*α* (*r* = 0.95), suggesting systemic effects in this model of allergic airway inflammation (Figure 5(b)).

### 3.9. Low Expression of Nlrp3 and IL-1*β* in Normal Human Bronchial Epithelial Cultures and Up-regulation by LPS but Not by IFN-*γ*

**Figure 6:**
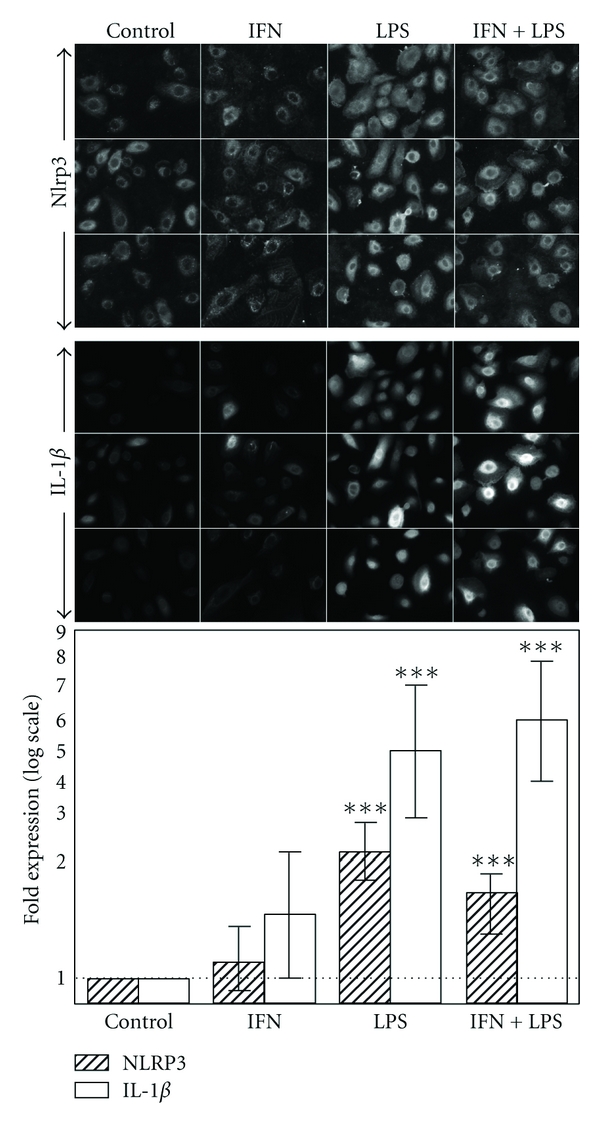
Induction of IL-1*β* and Nlrp3 protein expression in primary cell cultures of normal human bronchial epithelium by proinflammatory stimulus. Normal human bronchial epithelial (NHBE) cells were cultured without stimuli (control), or in presence of 50 ng/mL IFN-gamma (IFN), or 5 ug/mL E coli lipopolysaccharide (LPS), or their combination (IFN + LPS). Top and middle: fluorescence images of NHBE dual-labelled for intracellular Nlrp3 and IL-1*β*. Bottom: quantitation of fluorescence intensity by ImageJ software showing statistically significant increase (*P* < 0.05), when normalized to untreated controls, of both Nlrp3 and IL-1*β* in cells stimulated with LPS, or IFN + LPS combination. Error bars represent SEM. Expression of NRLP3 and IL1*β* was highly correlated within individual cells (*r* = 0.73, Spearman Rank correlation). Images are representative of  2 experiments with 30–50 cells analysed for each treatment.

 Commercially available passage 4 primary cultured normal human bronchial epithelial (NHBE) cells were studied for expression of the Nlrp3 and IL-1*β* proteins, before and after priming with LPS (5 ug/mL) or IFN-*γ* (50 ng/mL). Nlrp3 and IL-1*β* proteins were quantified in the same cell by dual-labelling with the respective antibodies (see methods) and using imageJ software to quantify fluorescence intensities. Between 26 and 51 cells (pooled from three randomly acquired images for each treatment) were analysed simultaneously for both proteins. Cells grown either in the absence of both agents or with IFN-*γ* alone expressed low levels of intracellular Nlrp3 and IL-1*β* as detected by immunofluorescence (and quantified using ImageJ software). Overnight stimulation with LPS or LPS plus IFN-*γ* resulted in significant increase (*P* < 0.05) of both Nlrp3 (~2 fold) and IL-1*β* (4-5 fold, Figure 6).

## 4. Discussion

This study provides the first evidence for presence of precursor components of the NRLP3 inflammasome in normal murine airway epithelium and some other resident or inflammatory airway cells. It also shows qualitative changes in the distribution of these markers, including appearance of active caspase-1, during airway inflammation in the acute mouse OVA model. Further studies are warranted to establish the functional significance of inflammasome activation as well as links between inflammasome regulation and other known mechanisms in asthma such as Th2 responses, IgE production, and its fixation on innate immune cell surfaces, eosinophilia, and airway tissue remodelling.

Our findings suggest a role for inflammasomes in innate immune responses of the airway epithelium and reinforce the hypothesis that airway epithelium plays a sentinel role in the innate immune response to inhaled microbes, allergens, and other pathogens. In particular, we have shown that the components necessary for mounting of a rapid protective inflammatory response via inflammasome activation are all expressed in the murine airway epithelium prior to inflammatory stimulation. Of interest, IL-1*β* and IL-18 were both expressed intracellularly within the normal murine airway epithelium. A similar expression of IL-1*β* was detected in rat lung epithelium tissue (data not shown). The failure to detect IL-1*β* in the circulation of the control mice suggests either that airway epithelium contributes very little to blood IL-1*β* levels or that signals leading to release of IL-1*β* (and probably also IL-18) are absent from normal airway epithelium. Our finding of intracellular IL-1*β* and IL-18 is therefore in keeping with the hypothesis that the airway epithelium is an important sentinel in the innate immune response and is primed for a rapid response when exposed to danger signals but has yet to undergo inflammasome activation.

Studies in cells of monocytic lineage have shown that to mount a proinflammatory response, the inflammasome pathway works in synergy with receptors for pathogen-associated molecular patterns (PAMPs) and danger-associated molecular patterns (DAMPs), the best characterized of which are TLRs. Thus, cell lines in their normal state usually do not express precursors of IL-1*β* and IL-18; production, maturation, and extracellular release of these potent proinflammatory cytokines require at least two signals [7]. Ligation of TLRs leads to enhanced transcription of the IL-1*β* gene and accumulation of an immature form of IL-1*β* in the cytoplasm. Stimulation with substances such as extracellular ATP or uric acid leads to a second signal that results in caspase-1 activation, processing of pro-IL-1*β*, and release of the mature cytokine. Using antibodies that detect the presence of neoepitopes in the caspase-1 subunits which become accessible only upon cleavage/activation of the proenzyme and a blocking peptide, we were able to provide qualitative evidence for presence of active caspase-1 in the airway epithelium of the OVA-treated mice.

We also found, in these OVA-treated mice, a potentially interesting and novel phenomenon of subcellular translocation for epithelial IL-1*β*, IL-18, and caspase-1, whereby these molecules were seen to be concentrated at the epithelial apical surface and apparently shed into the airway lumen in the form of cell bodies. The significance of this is unclear but may provide a mechanism for release of inflammasome-relevant molecules into the mucosa. IL-1*β* and IL-18 are proteins without a signal sequence [24]. The precursors accumulate in the cytosol following translation and a second signal is required for caspase-1-mediated cleavage of the precursors to the mature form that is released from the cell. Various mechanisms for release of the active IL-1 cytokines (and caspase-1, itself) have been postulated, including preexport into secretory lysosomes or encapsulation in microvesicles or exosomes [25]. Evidence for release as cell bodies or smaller vesicles has been provided by studies in monocytes [24, 26], microglia [27], and dendritic cells [28]. The clinical relevance of the luminal shedding of the IL-1 cytokines is yet to be explored.

Our findings support the notion that the airway epithelium can itself mount a proinflammatory response via activation of its inflammasome complexes and consequent release of IL-1*β* and IL-18. We were able to detect high levels of IL-1*β* in the circulation but not in the BALF. The reason for failure to detect the IL-1-like cytokines in BALF may be a consequence of dilution of apical secretions during our lavage procedure (total wash volume was 3 mL) or may be because the time interval between final OVA challenge and BALF collection (22 hr) resulted in missing the peak cytokine response. Relevant to this, 4 of the 8 OVA-treated mice had high levels of IL-1*β* and TNF-*α* in their serum while the other 4 mice (like all of the control mice) had very low levels of both cytokines, despite all 8 OVA-treated mice having high levels of airway inflammation as evidenced by eosinophilia in BALF and tissues as well as epithelial swelling and cell death. Our immunofluorescence studies also suggested that other potential sources of IL-1-like cytokines are eosinophils, alveolar macrophages, type II alveolar cells, and endothelium.

Relevant to our finding of NRLP3 in the normal mouse bronchiolar epithelium is the recent observation reporting NRLP3 and two other NLRs (NOD1 and NOD2) in upper airway human tissues including normal nasal mucosa, nasal polyps, tonsils, and adenoids [29]. It is likely therefore that inflammasome components are present along the entire length of the airways and constitute a front-line defence. Their involvement in allergic and inflammatory diseases of the airways such as chronic rhinosinusitis, rhinitis, and asthma warrants further study.

A major limitation with studies of the whole lung is that the lung is a complex organ comprising a number of tissues. During airway inflammation, there is also substantial infiltration of inflammatory cells, especially eosinophils. It is therefore difficult to interpret changes in gene expression at the level of a single cell type in the lung. Because of this we were driven from the start at establishing the techniques to show the presence of inflammasome components in specific cell types of the lung at a histochemical level. We think this is a major advantage of our study.

Our initial attempt to look at changes in expression of inflammasome-related genes in the context of murine airway inflammation showed a large increase in expression of the caspase inhibitor and inflammasome-binding protein NAIP as well as decreases in expression of IL-18 and NRLP3. Decreases in NRLP3 and IL18 gene expression during airway inflammation may indicate the existence of a negative feedback mechanism. Since NAIP is also able to substitute for NRLP3 and other NRLs in formation of the inflammasome complex [30], there may be a switch from NRLP3 to NAIP as a result of the inflammation. Discordance between mRNA and protein expression of P_2_X_7_ receptor, an important up-stream regulator of the NRLP3 inflammasome, has also been reported in inflamed intestinal epithelium from patients with inflammatory bowel disease [31]. In that study, in which P_2_X_7_ receptor engagement was required for production and release of IL-1*β*, mRNA levels for P_2_X_7_ receptor were increased while protein levels were strongly decreased. The decrease in protein levels was much greater in patients with active disease compared to those with quiescent disease and correlated with the degree of polymorphonuclear infiltration into the epithelium (transepithelial migration). The authors speculated that the decrease in protein expression may help to protect the intestinal epithelial cells from excessive activation of P_2_X_7_ receptor and subsequent cell death during the neutrophil transmigration. Eosinophilic infiltration into the airway epithelium is also important in the pathogenesis of asthma [32]. Downregulation of NRLP3 protein may have a similar protective role against inflammation-associated cell death during episodes of asthma.

Dissection of the mechanisms involved in airway epithelial inflammasome regulation and activation will require the use of *in vitro* airway epithelial cultures. We have shown that the presence of NRLP3 and Il-1*β* proteins in the commercially available normal human bronchial epithelial primary cells and the 2–5-fold upregulation of these proteins following priming with LPS indicates involvement of TLR4 in the priming. Another proinflammatory stimulus INF*γ* which acts via a distinct signalling pathway was ineffective.

In conclusion, we have demonstrated the presence of precursor components of the NRLP3 inflammasome in healthy murine airway epithelium as well as changes in the subcellular distribution of these components and appearance of active caspase-1 in the epithelium of inflamed airways. At this stage we know little about the extent to which the innate immune system and inflammasome activation in particular exert protective or detrimental effects in asthma. Further studies using both lung biopsies from patients and mechanistic studies in polarized cultures of human airway epithelium are warranted and may reveal new insight into the disease mechanism and additional targets for therapeutic and/or diagnostic applications in asthma.

## Figures and Tables

**Table 1:** Ovalbumin-induced changes at mRNA level in mouse lung.

Gene product*	Relevance	Ratio** OVA/SAL	*P* value***
Ccl12	Chemokine of CC family which attracts eosinophils and monocytes	10.64	0.008
Ccl7	Chemokine of CC family which attracts monocytes and regulates macrophage function	8.98	0.018
NAIP 1 (neuronal apoptosis inhibitor protein)	Endogenous caspase inhibitor which binds to the inflammasome in neuronal and nonneuronal cell types	8.57	0.000
Cxcl3	CXC chemokine which controls migration and adhesion of monocytes	8.31	0.016
IL-6	Th2 cytokine which promotes airway inflammation in mice	3.08	0.050
IL-33	IL-1 like cytokine which mediates eosinophilia in mouse asthma model	2.54	0.001
ASC	Adaptor protein which recruits caspase-1 to the NLRP3 complex	2.11	0.001
Caspase 1	When activated, it processes pro-IL1b and pro-IL18 to mature cytokines	2.07	0.000
Aim2 (absent in melanoma gene 2)	Component of the AIM2 Inflammasome which activates caspase-1 independent of NLR proteins	2.00	0.002
NLRP1a	Component of the NLRP1 Inflammasome which activates caspases 1 and 5	1.07	0.658
NLRC4 (IPAF)	Component of the NLRP4 Inflammasome which mediates response to *S. typhimurium *	0.90	0.434
IL-1*β*	Member of IL-1 family of cytokines	0.48	0.003
NLRP3	Component of the NLRP3 Inflammasome which activates caspases 1	0.28	0.000
IL-18	Member of IL-1 family of cytokines	0.09	0.000

*Data from a superarray experiment on lung RNA from 8 SAL and 8 OVA-treated mice. Genes relevant to the asthma/inflammasome pathways are shown. **Fold up- or downregulation for OVA-treated mice relative to saline-treated mice was determined by the ΔΔCt method. Ratios are in decreasing order; ratios >1 represent increased expression with airway inflammation and ratios <1 represent decreased expression with airway inflammation. Upregulation of Caspase-1 and downregulation of IL-1*β* were also confirmed by an “in-house” RT-PCR assay. ***Student *T*-test.

## Abbreviations

Abs: Antibodies
ASC: Apoptosis-associated speck-like protein containing a caspase-recruitment domain
ATII: Type 2 alveolar cells
BALF: Bronchoalveolar lavage fluid
DAMP: Danger-associated molecular pattern
HBSS: Hanks balanced salt solution
IL-1*β*: Interleukin-1*β*
IFN-*γ*: Interferon *γ*
LPS: lipopolysaccharide
NHBE: Normal human bronchial epithelial cells
NAIP: Neuronal Apoptosis Inhibitor Protein
NLR: NOD-like receptor
NOD: Nucleotide-binding oligomerization domain
OVA: Ovalbumin
PAMP: Pathogen-associated molecular pattern
PBS: phosphate-buffered saline
SAL: Saline
SP-D: Surfactant protein D
TBST: Tris-buffered saline containing Tween 20
TLRs: Toll-like receptors
TNF-*α*: Tumour necrosis factor-*α*
XIAP: X-linked inhibitor of apoptosis protein.

## References

[1] Adams RJ, Wilson DH, Appleton S (2003). Underdiagnosed asthma in South Australia. *Thorax*.

[2] Hogan SP, Mould A, Kikutani H, Ramsay AJ, Foster PS (1997). Aeroallergen-induced eosinophilic inflammation, lung damage, and airways hyperreactivity in mice can occur independently of IL-4 and allergen-specific immunoglobulins. *Journal of Clinical Investigation*.

[3] Temelkovski J, Hogan SP, Shepherd DP, Foster PS, Kumar RK (1998). An improved murine model of asthma: selective airway inflammation, epithelial lesions and increased methacholine responsiveness following chronic exposure to aerosolised allergen. *Thorax*.

[4] Foster PS, Yang M, Herbert C, Kumar RK (2002). CD4^+^ T-lymphocytes regulate airway remodeling and hyper-reactivity in a mouse model of chronic asthma. *Laboratory Investigation*.

[5] Sims JE, Smith DE (2010). The IL-1 family: regulators of immunity. *Nature Reviews Immunology*.

[6] Hoffman HM, Wanderer AA (2010). Inflammasome and IL-1*β*-mediated disorders. *Current Allergy and Asthma Reports*.

[7] Tschopp J, Schroder K (2010). NLRP3 inflammasome activation: the convergence of multiple signalling pathways on ROS production?. *Nature Reviews Immunology*.

[8] Yilmaz O, Sater AA, Yao L, Koutouzis T, Pettengill M, Ojcius DM (2010). ATP-dependent activation of an inflammasome in primary gingival epithelial cells infected by Porphyromonas gingivalis. *Cellular Microbiology*.

[9] Schroder NWJ (2009). The role of innate immunity in the pathogenesis of asthma. *Current Opinion in Allergy and Clinical Immunology*.

[10] De Amici M, Puggioni F, Casali L, Alesina R (2001). Variations in serum levels of interleukin (IL)-1*β*, IL-2, IL-6, and tumor necrosis factor-*α* during specific immunotherapy. *Annals of Allergy, Asthma and Immunology*.

[11] Mahajan B, Vijayan VK, Agarwal MK, Bansal SK (2008). Serum interleukin-1*β* as a marker for differentiation of asthma and chronic obstructive pulmonary disease. *Biomarkers*.

[12] Broide DH, Lotz M, Cuomo AJ, Coburn DA, Federman EC, Wasserman SI (1992). Cytokines in symptomatic asthma airways. *Journal of Allergy and Clinical Immunology*.

[13] Ayanoglu G, Desai B, Fick Jr. RB (2011). Modelling asthma in macaques: longitudinal changes in cellular and molecular markers. *European Respiratory Journal*.

[14] Sousa AR, Trigg CJ, Lane SJ (1997). Effect of inhaled glucocorticoids on IL-1*β* and IL-1 receptor antagonist (IL- 1ra) expression in asthmatic bronchial epithelium. *Thorax*.

[15] Tsukagoshi H, Sakamoto T, Xu W, Barnes PJ, Chung KF (1994). Effect of interleukin-1*β* on airway hyperresponsiveness and inflammation in sensitized and nonsensitized Brown-Norway rats. *Journal of Allergy and Clinical Immunology*.

[16] Zhang Y, Xu CB, Cardell LO (2009). Long-term exposure to IL-1*β* enhances Toll-IL-1 receptor-mediated inflammatory signaling in murine airway hyperresponsiveness. *European Cytokine Network*.

[17] Ando M, Shima M (2007). Serum interleukins 12 and 18 and immunoglobulin E concentrations and allergic symptoms in Japanese schoolchildren. *Journal of Investigational Allergology and Clinical Immunology*.

[18] Patil SP, Wisnivesky JP, Busse PJ, Halm EA, Li X-M (2011). Detection of immunological biomarkers correlated with asthma control and quality of life measurements in sera from chronic asthmatic patients. *Annals of Allergy, Asthma and Immunology*.

[19] Tanaka H, Miyazaki N, Oashi K (2001). IL-18 might reflect disease activity in mild and moderate asthma exacerbation. *Journal of Allergy and Clinical Immunology*.

[20] Gasse P, Riteau N, Charron S (2009). Uric acid is a danger signal activating NALP3 inflammasome in lung injury inflammation and fibrosis. *American Journal of Respiratory and Critical Care Medicine*.

[21] Idzko M, Hammad H, Van Nimwegen M (2007). Extracellular ATP triggers and maintains asthmatic airway inflammation by activating dendritic cells. *Nature Medicine*.

[22] Holgate ST (2010). A look at the pathogenesis of asthma: the need for a change in direction. *Discovery Medicine*.

[23] Truong-Tran AQ, Ruffin RE, Foster PS (2002). Altered zinc homeostasis and caspase-3 activity in murine allergic airway inflammation. *American Journal of Respiratory Cell and Molecular Biology*.

[24] Rubartelli A, Cozzolino F, Talio M, Sitia R (1990). A novel secretory pathway for interleukin-1*β*, a protein lacking a signal sequence. *EMBO Journal*.

[25] Eder C (2009). Mechanisms of interleukin-1*β* release. *Immunobiology*.

[26] Sarkar A, Mitra S, Mehta S, Raices R, Wewers MD (2009). Monocyte derived microvesicles deliver a cell death message via encapsulated caspase-1. *PLoS One*.

[27] Bianco F, Pravettoni E, Colombo A (2005). Astrocyte-derived ATP induces vesicle shedding and IL-1*β* release from microglia. *Journal of Immunology*.

[28] Pizzirani C, Ferrari D, Chiozzi P (2007). Stimulation of P2 receptors causes release of IL-1*β*-loaded microvesicles from human dendritic cells. *Blood*.

[29] Månsson A, Bogefors J, Cervin A, Uddman R, Cardell LO (2011). NOD-like receptors in the human upper airways: a potential role in nasal polyposis. *Allergy*.

[30] Martinon F (2007). Orchestration of pathogen recognition by inflammasome diversity: variations on a common theme. *European Journal of Immunology*.

[31] Cesaro A, Brest P, Hofman V (2010). Amplification loop of the inflammatory process is induced by P2X_7_R activation in intestinal epithelial cells in response to neutrophil transepithelial migration. *American Journal of Physiology*.

[32] Persson C, Uller L (2010). Transepithelial exit of leucocytes: inflicting, reflecting or resolving airway inflammation?. *Thorax*.

[33] Besnard AG, Guillou N, Tschopp J (2011). NLRP3 inflammasome is required in murine asthma in the absence of aluminum adjuvant. *Allergy*.

